# HIV infection in patients with sexually transmitted infections in Zimbabwe – Results from the Zimbabwe STI etiology study

**DOI:** 10.1371/journal.pone.0198683

**Published:** 2018-06-11

**Authors:** Peter H. Kilmarx, Elizabeth Gonese, David A. Lewis, Z. Mike Chirenje, Beth A. Tippett Barr, Ahmed S. Latif, Lovemore Gwanzura, H. Hunter Handsfield, Anna Machiha, Owen Mugurungi, Cornelius A. Rietmeijer

**Affiliations:** 1 Division of Global HIV and TB, Centers for Disease Control and Prevention, Atlanta, Georgia, United States of America; 2 Division of Global HIV and TB, U.S. ‎Centers for Disease Control and Prevention, Harare, Zimbabwe; 3 Western Sydney Sexual Health Centre, Marie Bashir Institute for Infectious Diseases and Biosecurity, Sydney Medical School—Westmead, University of Sydney, Sydney, Australia; 4 Department of Obstetrics, Gynecology & Reproductive Sciences, College of Health Sciences, University of Zimbabwe, Harare, Zimbabwe; 5 Public Health Consultant, Calamvale, Brisbane, Australia; 6 Department of Medical Laboratory Sciences, College of Health Sciences, University of Zimbabwe, Harare, Zimbabwe; 7 Department of Medicine, University of Washington, Seattle, Washington, United States of America; 8 Zimbabwe Ministry of Health and Child Care, Harare, Zimbabwe; 9 Surveillance, Evaluation, Assessment, and Monitoring (SEAM) Project, Department of Community Medicine, University of Zimbabwe, College of Medicine, Harare, Zimbabwe; 10 Colorado School of Public Health, University of Colorado Denver, Colorado, United States of America; 11 Rietmeijer Consulting, Denver, Colorado, United States of America; London School of Hygiene and Tropical Medicine, UNITED KINGDOM

## Abstract

**Background:**

HIV and other sexually transmitted infections (STI) frequently co-occur. We conducted HIV diagnostic testing in an assessment of the etiologies of major STI syndromes in Zimbabwe.

**Methods:**

A total of 600 patients were enrolled at six geographically diverse, high-volume STI clinics in Zimbabwe in 2014–15: 200 men with urethral discharge, 200 women with vaginal discharge, and 100 men and 100 women each with genital ulcer disease (GUD). Patients completed a questionnaire, underwent a genital examination, and had specimens taken for etiologic testing. Patients were offered, but not required to accept, HIV testing using a standard HIV algorithm in which two rapid tests defined a positive result.

**Results:**

A total of 489 participants (81.5%) accepted HIV testing; 201 (41.1%) tested HIV-1-positive, including 16 (11.9%) of 134 participants who reported an HIV-negative status at study enrollment, and 58 (28.2%) of 206 participants who reported their HIV status as unknown. Of 147 who self-reported being HIV-positive at study enrollment, 21 (14.3%) tested HIV negative. HIV infection prevalence was higher in women (47.3%) than in men (34.8%, p<0.01), and was 28.5% in men with urethral discharge, 40.5% in women with vaginal discharge, 45.2% in men with GUD, and 59.8% in women with GUD (p<0.001).

**Conclusions:**

The high prevalence of HIV infection in STI clinic patients in Zimbabwe underscores the importance of providing HIV testing and referral for indicated prevention and treatment services for this population. The discrepancy between positive self-reported and negative study HIV test results highlights the need for operator training, strict attention to laboratory quality assurance, and clear communication with patients about their HIV infection status.

## Introduction

HIV and other sexually transmitted infections (STIs) are epidemiologically linked. HIV infection increases susceptibility to or infectiousness with many STIs, and, vice versa, many STIs increase susceptibility to and infectiousness with HIV infection [1]. Integrated health services are recommended for people at risk for or with either type of infection. Enhanced prevention, screening, and treatment of STIs are recommended for people living with HIV infection [2], and, vice versa, enhanced HIV prevention, testing, and treatment are recommended for individuals with STIs [3]. HIV testing, in particular, is the gateway to either HIV prevention services, such as male circumcision or use of HIV pre-exposure prophylaxis, for those who test HIV negative or to HIV care and treatment services for those who test HIV positive [4].

We conducted HIV diagnostic testing in an assessment of the etiologies of the major STI syndromes in patients visiting a geographically diverse set of STI clinics Zimbabwe in 2014–15 [5]. In the current paper, we report prevalence and risk factors for HIV infection in the study population, including associations of HIV infection with other STIs. Other studies in the region [6,7,8,9] have found high rates of HIV infection in STI patients, highlighting the importance of HIV diagnostic testing and linkage to prevention or treatment services for these populations.

Ensuring the quality of HIV testing is a critical component of scaling up HIV prevention, care, and treatment [10]. Both false negative and false positive test results may occur, as well as miscommunication with patients, which, respectively, could result in either failure to treat HIV infection or inappropriate antiretroviral treatment in HIV-uninfected patients. In this paper, we also report on participants’ self-reported HIV infection status and compared it to their study test results.

## Methods

The study methods have been reported elsewhere [5], and the full study protocol and questionnaires are available online [11]. Briefly, the Zimbabwe STI Etiology Study was designed to correlate laboratory-documented etiology with syndromic management guidelines for STI management. We enrolled 200 men presenting with urethral discharge, 200 women presenting with vaginal discharge, and 100 men and 100 women each presenting with genital ulcers at six geographically diverse, high-volume STI clinics in Zimbabwe. Participants were enrolled consecutively. The sample size was sufficient to detect a difference between equal size groups in HIV prevalence of 40% vs. 52% (odds ratio = 1.7). After giving written informed consent, patients completed a standardized paper-based questionnaire administered by trained study personnel, underwent a standardized genital examination, and had urethral, vaginal, urine, and/or genital ulcer specimens collected for etiologic testing. A blood sample was taken by venipuncture for HIV and syphilis serologic testing, for which separate written consent was obtained. All patient specimens were kept refrigerated after collection and shipped in a cooler box with cooling packs by courier the same day or overnight to the receiving laboratory at Wilkins Hospital in Harare where all samples were kept refrigerated until further processing. The following tests were conducted at the receiving laboratory: Gram-staining and reading of the air-dried smears, HIV testing, non-treponemal testing by RPR, and treponemal testing by TPHA. HIV rapid testing followed the algorithm promoted by the Zimbabwe Ministry of Health and Child Care (MOHCC) and routinely employed nationwide: an initial test by First Response HIV1-2-O (Premier Medical Corporation, Daman, India); if positive, a confirmatory Alere Determine HIV1/2 test (Alere Inc. Waltham, MA, USA); and, in case of discrepancy between these results, an INSTI HIV1/HIV2 test (bioLytical Laboratories Inc. Richmond, BC, Canada) as tie breaker.

Urine and vaginal samples of all patients (including patients with genital ulcer disease) were shipped for testing for *N*. *gonorrhoeae* and *C*. *trachomatis* by nucleic acid amplification testing in parallel in two laboratories 1) using ProbeTec (Becton Dickinson, Franklin Lakes, NJ, USA) at the University of Zimbabwe/University of California San Francisco laboratory at the University of Zimbabwe School of Medicine and 2) using GeneXpert (Cepheid, Sunnyvale, CA, USA) at the Flow cytometry laboratory in Harare. Genital discharge and ulcer samples were stored in a -70°F freezer and batched for shipment to the STI reference laboratory at the National Institute for Communicable Diseases (NICD) in Johannesburg, South Africa. Using an in-house developed multiplex polymerase chain reaction test procedure, discharge specimens were tested for *N*. *gonorrhoeae*, *C*. *trachomatis*, *T*. *vaginalis*, and *M*. *genitalium*. A test was considered positive if the result of any of the three tests for *N*. *gonorrhoeae* and *C*. *trachomatis* was positive. An in-house developed multiplex polymerase chain reaction test procedure was also used to test ulcer specimens for T. *pallidum*, *H*. *ducreyi*, *C*. *trachomatis—*lymphogranuloma venereum serovars L1-3, herpes simplex virus (HSV)-1, and HSV-2.

Data were analyzed using SAS software (Cary, NC, USA). Statistical analyses included Chi-Square and Fisher’s Exact Test for categorical data and Student’s T-test for continuous variables. Sites were purposively selected and not statistically representative of the STI clinics, therefore, we did not account for possible site clustering in the analysis. The protocol was approved by the institutional review boards of the University of Zimbabwe, the Zimbabwe Medical Research Council, and the U.S. Centers for Disease Control and Prevention.

## Results

Study enrollment began in June 2014 and was completed in April 2015. Six hundred participants were enrolled, 200 in each of the syndrome categories. The participants’ mean (median) age was 28.6 (27) years; enrolled women were somewhat younger (mean age 27.7 years) than enrolled men (29.4 years, p<0.01). The mean number of sex partners in the prior three months was 2.1 (median 1), 24.5% reported more than one partner and 14.5% reported engaging in commercial sex during the same interval. Among participants who reported a main sex partner, 24.2% reported using condoms at last sex with that partner, whereas 40.9% reported condom use at last sex with a non‐main partner. By self‐report, 29.2% of all study participants were HIV positive, 29.6% were HIV negative, and 41.1% reported an unknown HIV infection status.

A total of 489 (81.5%) participants accepted HIV testing; all further results refer to this subset of patients. Those who accepted and declined HIV testing were similar with regard to age and gender (data not shown). Those with discharge syndromes were more likely to decline HIV testing (males– 24.5%, females– 21.0%) compared with those with genital ulcers (males—7.0%, females– 13.0%, p <0.001). Those who thought they were HIV negative were somewhat more likely to decline (23.4%) compared with those who thought they were HIV positive (16.9%) or were unsure (16.1%, p = 0.13).

Associations of HIV infection with demographic characteristics and risk behaviors are presented in Table 1. Overall, 41.1% tested HIV-1-positive, including 16 (11.9%) of 134 participants who reported an HIV-negative status at study enrollment, and 59 (28.4%) of 208 participants who reported an HIV-unknown status. These prevalences were similar in men and women. Among the 134 participants who reported that they were HIV uninfected, we compared the 16 HIV-infected participants with the 118 who were uninfected. We did not find any significant association with the sex of the participant, age group, the number of reported sex partners, engaging in sex work (self or partner), or condom use with main or casual partners (data not shown). All HIV infections were HIV type 1; none was HIV type 2. Of the 147 who self-reported being HIV-positive at study enrollment, 21 (14.3%) tested HIV negative. This misreporting was more common in men (23.3%) than in women (8.0%, p < .009). Women had a higher HIV infection prevalence (47.3%) than men (34.8%, p = 0.005). HIV prevalence did not vary significantly by study site or by language-ethnic group (data not shown). Those with HIV infection were significantly older (mean 30.5, median 30 years) compared to their HIV-negative counterparts (mean 27.3, median 26 years, p<0.0001). HIV infection was not associated with reporting more than one sexual partner, but was associated with engaging in sex work in the previous three months in women. Those who reported condom use at last sex with both main and non‐main partners were more likely to be HIV infected. This increased HIV infection prevalence with reported condom use was in both men and women (data not shown).

**Table 1:** Demographic characteristic and risk behavior associations with prevalence of HIV-1 infection in sexually transmitted infection clinic patients, Zimbabwe– 2014–2015.

	HIV positive–n (%)	HIV negative–n (%)	OR (95% CI)
Overall	201 (41.1)	288 (58.9)	
Self-perceived HIV infection status			
Positive	126 (85.7)	21 (14.3)	**44.3 (22.0–88.9)**
Negative	16 (11.9)	118 (88.1)	Ref
Unsure	59 (28.4)	149 (71.6)	**2.92 (1.60–5.34)**
Sex			
Male	85 (34.8)	159 (65.2)	Ref
Female	116 (47.3)	129 (52.7)	**1.68 (1.17–2.42)**
Age, years			
15–24	47 (28.0)	121 (72.0)	Ref
25–34	107 (45.7)	127 (54.3)	**2.17 (1.42–3.31)**
35+	47 (54.0)	40 (46.0)	**3.03 (1.76–5.19)**
Number sex partners			
0–1	161 (42.6)	217 (57.4)	Ref
2+	40 (36.0)	71 (64.0)	0.76 (0.49–1.18)
Sex worker or sex worker partner			
Men, sex worker partner			
Yes	15 (28.8)	37 (71.2)	0.71 (0.36–1.38)
No	70 (36.5)	122 (63.5)	Ref
Women, sex worker			
Yes	18 (72.0)	7 (28.0)	**3.2 (1.29–7.97)**
No	98 (44.5)	122 (55.5)	Ref
Condom use at last sex with main partner			
Yes	66 (54.5)	55 (45.5)	**2.05 (1.35–3.12)**
No	131 (36.9)	224 (63.1)	Ref
Condom use at last sex with non-main partner			
Yes	88 (47.6)	97 (52.4)	**1.60 (1.08–2.36)**
No	88 (36.2)	155 (63.8)	Ref

OR, odds ratio; CI, confidence interval.

**Fig 1:**
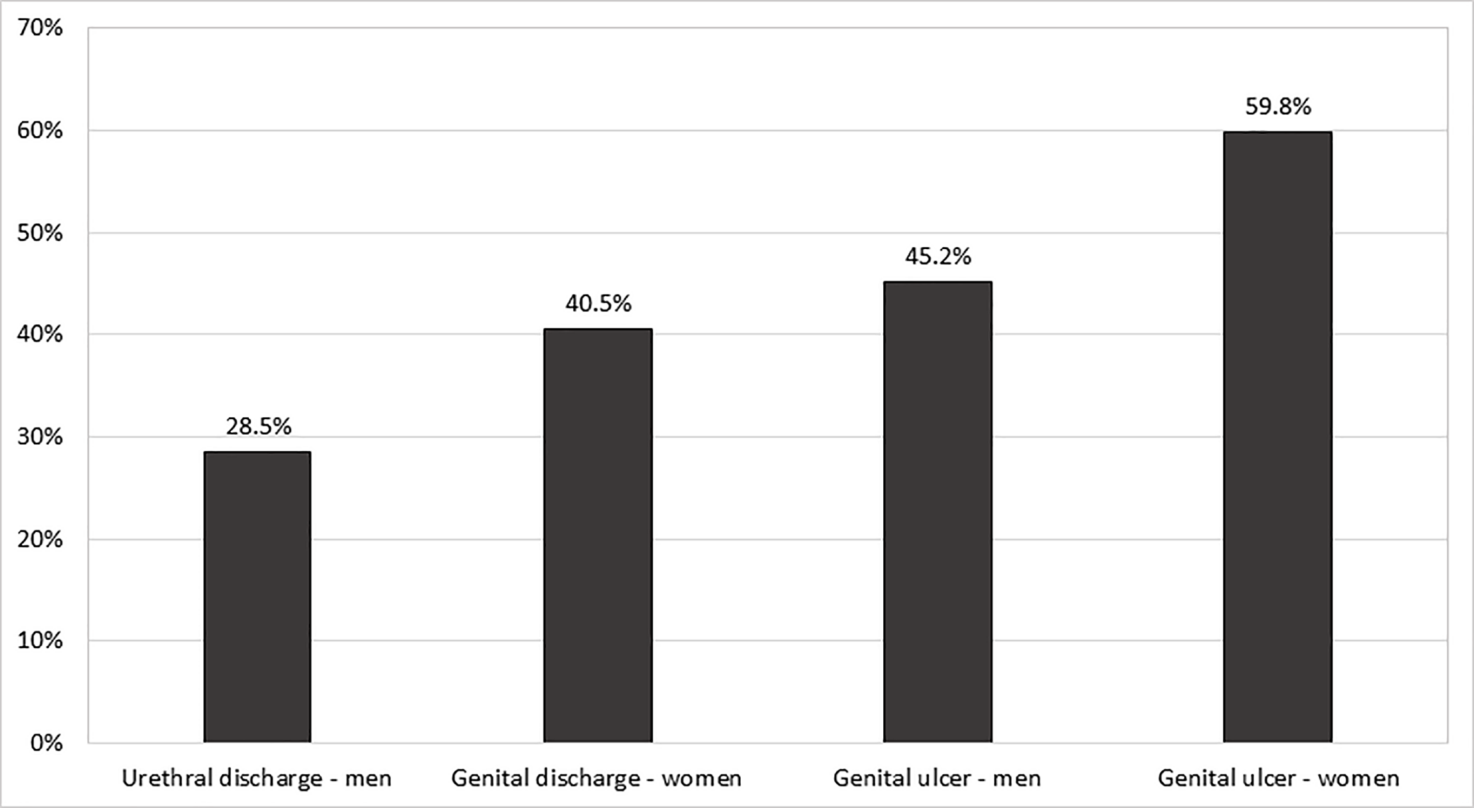
Prevalence of HIV-1 infection in sexually transmitted infection (STI) clinic patients, by sex and STI syndrome, Zimbabwe– 2014–2015.

Associations of HIV infection with STIs are presented in Table 2. HIV prevalence varied significantly across the different STI syndromes (Fig 1), with the lowest prevalence in men with urethral discharge (28.5%) and the highest prevalence in women with genital ulcers (59.8%, p<0.001). HIV prevalence was associated with gonococcal infection in women, but not in men. In both men and women, HIV infection was somewhat less common in those with chlamydial infection, but this was not statistically significant. Among women with vaginal discharge, there was no association between HIV prevalence and a Gram stain diagnosis of bacterial vaginosis or yeast infection. In both men and women with genital ulcer disease, HIV prevalence was strongly associated with herpes simplex virus (HSV-2) ulcers. There was a non-significant association between HIV prevalence and genital ulcers caused by *T*. *pallidum* in women, but not in men. HIV infection was associated with positivity with both non-treponemal and treponemal syphilis blood tests.

**Table 2:** Sexually transmitted infection (STI) associations with prevalence of HIV-1 infection in STI clinic patients, Zimbabwe– 2014–2015.

	HIV positive–n (%)	HIV negative–n (%)	OR (95% CI)
Overall	201 (41.1)	288 (58.9)	
Presenting STI syndrome			
Male discharge	43 (28.5)	108 (71.5)	Ref
Female discharge	64 (40.5)	94 (59.5)	**1.71 (1.06–2.75)**
Male ulcer	42 (45.2)	51 (54.8)	**2.07 (1.21–3.55)**
Female ulcer	52 (59.8)	35 (40.2)	**3.73 (2.14–6.50)**
*N*. *gonorrhoeae* infection			
Men			
Positive	40 (33.3)	80 (66.7)	0.88 (0.52–1.49)
Negative	45 (36.3)	79 (63.7)	Ref
Women			
Positive	39 (62.9)	23 (37.1)	**2.33 (1.29–4.22)**
Negative	77 (42.1)	106 (57.9)	Ref
*C*. *trachomatis* infection			
Men			
Positive	14 (25.5)	41 (74.5)	0.57 (0.29–1.11)
Negative	71 (37.6)	118 (62.4)	Ref
Women			
Positive	16 (39.0)	25 (61.0)	0.67 (0.34–1.32)
Negative	100 (49.0)	104 (51.0)	Ref
Bacterial vaginosis (women)			
Positive	15 (38.5)	24 (61.5)	0.97 (0.44–2.15)
Negative	29 (39.2)	45 (60.8)	Ref
Vaginal candidiasis (women)			
Positive	12 (38.7)	19 (61.3)	0.98 (0.41–2.32)
Negative	29 (39.2)	45 (60.8)	Ref
Herpes Simplex Virus ulcer swab			
Men			
Positive	22 (61.1)	14 (38.9)	**2.91 (1.23–6.89)**
Negative	20 (35.1)	37 (64.9)	Ref
Women			
Positive	26 (76.5)	8 (23.5)	**3.38 (1.29–8.80)**
Negative	26 (49.1)	27 (50.9)	Ref
*T*. *pallidum* ulcer swab			
Men			
Positive	7 (41.2)	10 (58.8)	0.82 (0.28–2.38)
Negative	35 (46.1)	41 (53.9)	Ref
Women			
Positive	10 (83.3)	2 (16.7)	3.93 (0.81–19.17)
Negative	42 (56.0)	33 (44.0)	Ref
Rapid plasma reagin (RPR) test			
Positive	29 (58.0)	21 (42.0)	**2.14 (1.18–3.88)**
Negative	172 (39.2)	267 (60.8)	Ref
Treponemal aemagglutination (TPHA) test			
Positive	43 (60.6)	28 (39.4)	**2.48 (1.47–4.17)**
Negative	137 (38.3)	221 (61.7)	Ref

OR, odds ratio; CI, confidence interval.

This study has limitations: The study sites were purposively chosen, hence the results cannot be generalized beyond the study sites. All HIV tests were conducted in the field without additional laboratory confirmation and so this could potentially account for misclassification of HIV sero-status.

## Discussion

In this study of 600 patients in STI clinics in Zimbabwe, there was a high overall HIV sero-prevalence of 41.1%. HIV infection prevalence was higher in women (47.3%), particularly those with genital ulcer (59.8%). HIV infection was common in those who reported a previously unknown infection status (28.4%) or previously testing HIV negative (11.9%). HIV prevalence in this STI study population was 2.8 times higher than in the overall adult population (14.7%) [12], but was similar to HIV infection prevalence in other STI study populations in the region [6,7,8,9].

This high HIV prevalence underscores the importance of HIV screening in STI clinic populations. Testing those with unknown or previously negative status will identify numerous previously undiagnosed cases, which represented 36.8% of those who tested HIV positive in this study. Linking HIV-infected persons to HIV care and treatment is essential. Where feasible, these services should be co-located with STI clinic services to facilitate linkage to care. Ensuring re-engagement in HIV care and antiretroviral treatment with known HIV-infected STI clinic patients who are not in care is also important, both for the health of the HIV-infected individual and to reduce the risk of onward HIV transmission. Partner notification and partner HIV testing are also recommended where feasible [4].

The high HIV prevalence in those who reported previously testing HIV negative suggests a high HIV incidence in this population, highlighting the critical importance of HIV prevention interventions for the HIV-uninfected individuals, including risk reduction counseling and provision of condoms, referral for circumcision for male clients, and, where available, use of antiretroviral pre-exposure prophylaxis.

There was a substantial discrepancy between self-reported HIV-infection status and study laboratory HIV test results. For those who reported having previously tested HIV negative and their study HIV test results were positive, this may represent incident HIV infection in the interval since their previous test or could be misreporting of the previous test result due to perceived stigma. A previous false negative test result is also possible, as is a false positive study test result, although this seems less likely in the context of a carefully conducted research study. A striking 14.3% of participants who reported that they were HIV positive actually tested HIV negative in the study. There may have been be some misreporting, e.g., if participants thought there was some additional study benefit for HIV-infected persons. Alternatively, participants may have misunderstood the study question, or their previous test results may have been miscommunicated. There is also the possibility of previous false positive test results. Finally, some participants may have had HIV-infected partners and therefore assumed they were infected, without actual laboratory confirmation. In any case, clinicians should be aware that serological confirmation is warranted for some patients who believe they have HIV. This finding reconfirms the need for regular staff training, strict attention to laboratory quality assurance [10], documentation of results, and clear communication with patients about their HIV infection status. We did not ascertain whether any HIV-uninfected participants were inappropriately using of antiretroviral medications, but this possibility highlights the need for reconfirmation of HIV infection prior to initiating treatment, especially with the implementation of “test and start” treatment programs [13].

In this STI clinic study population, HIV infection was not associated with having more than one partner or engaging in commercial sex in the prior three months and was positively associated with higher reported condom use. These seemingly paradoxical results may be due in part to “overmatching.” All study participants had a chief complaint of an STI syndrome, therefore even the HIV-uninfected participant group had high-risk sexual behaviors. Moreover, most of the HIV-infected participants (63.2%) had been previously diagnosed, so they may have already decreased their risk behavior, including unprotected sex, before study entry, or they reported reduced risk behavior due to social desirability bias. A practical implication of these findings is that HIV testing and other services should be offered to all STI clinic patients. There is no identifiable sub-group with truly low HIV infection rates and little to be gained in trying to target services.

The association of HIV infection with most STIs is likely due to sexual risk behavior increasing the risk of both HIV and other STI, as well as the potential for some STIs to increase susceptibility to HIV infection [11]. Conversely, HIV-related immunosuppression increases the likelihood of recurrence and persistence of genital ulcers caused by HSV-2 [11]. Because genital ulceration and other STIs increase infectiousness with HIV, screening and treatment of STIs in HIV-infected persons and routine testing and treatment for HIV in STI patients are important HIV prevention interventions [2,3]. To achieve these goals, increased access to point-of-care STI testing for those with and at risk for HIV infection is needed [14]. HIV infection, which was associated with older age, was not more frequent in those with chlamydial infection, which was more prevalent in younger participants (data not shown). We speculate that HIV infection may be a marker for sexual activity patterns associated with multiple infections with (or exposure to) *Chlamydia trachomatis*, with acquired immunity helping to explain the lack of association [15].

In conclusion, we found a high prevalence of HIV infection (41.1%) in STI clinic patients in Zimbabwe. This high prevalence, taken together with the mutual potentiation of transmission of HIV and other STIs, confirms the importance of providing high quality HIV testing and referral for appropriate prevention and treatment services in this setting for the benefit of both the individual and public health. Our findings support MOHCC recommendations to routinely recommend and undertake HIV screening in all patients seeking STI services in Zimbabwe including potential retesting of some patients who report already having HIV infection.

## Disclaimer

The content is solely the responsibility of the authors and does not necessarily represent the official views of the U.S. Centers for Disease Control and Prevention or the Zimbabwe Ministry of Health and Child Care.

## Supporting information

S1 File Study data. The study data are in the file.
(CSV)
